# Temporal mRNA Expression of Purinergic P2 Receptors in the Brain Following Cerebral Ischemia and Reperfusion: Similarities and Distinct Variations Between Rats and Mice

**DOI:** 10.3390/ijms26062379

**Published:** 2025-03-07

**Authors:** Siva Reddy Challa, Hunter Levingston, Casimir A. Fornal, Isidra M. Baker, Joseph Boston, Nidhi Shanthappa, Pavani Unnam, Jeffrey D. Klopfenstein, Krishna Kumar Veeravalli

**Affiliations:** 1 Department of Cancer Biology and Pharmacology, University of Illinois College of Medicine Peoria, Peoria, IL 61605, USA; siva09@uic.edu (S.R.C.); levings2@uic.edu (H.L.); cfornal@uic.edu (C.A.F.); ibaker5@uic.edu (I.M.B.); jboston2@illinois.edu (J.B.); punnam2@uic.edu (P.U.); jeffklop@ini.org (J.D.K.); 2 Department of Neurosurgery, University of Illinois College of Medicine Peoria, Peoria, IL 61605, USA; 3 Illinois Neurological Institute, OSF HealthCare, Peoria, IL 61603, USA; 4 Department of Pediatrics, University of Illinois College of Medicine Peoria, Peoria, IL 61605, USA; 5 Department of Neurology, University of Illinois College of Medicine Peoria, Peoria, IL 61605, USA

**Keywords:** stroke, ischemia, reperfusion, purinergic, receptor, expression, brain

## Abstract

Purinergic P2 receptors are crucial in energy utilization and cellular signaling, making them key targets for stroke therapies. This study examines the temporal mRNA expression of all P2 receptors in rats and mice. Both species exhibited a common subset of P2X and P2Y receptors with elevated expression following cerebral ischemia and reperfusion (I/R), highlighting conserved mechanisms across these species. The receptors with upregulated expression in both species were P2X3, P2X4, P2X7, P2Y2, and P2Y6. While these similarities were observed, notable differences in receptor expression emerged between rats and mice. Rats exhibited a broader receptor profile, with five additional receptors (P2X1, P2Y1, P2Y12, P2Y13, and P2Y14) significantly upregulated compared to only two receptors (P2X2 and P2Y4) in mice, highlighting species-specific regulation of receptor expression distinct from the shared receptors. Following cerebral I/R, P2Y12 was the most upregulated receptor in rats, while P2Y2 was the most upregulated in mice. These findings reveal both conserved and species-specific changes in P2 receptor expression following cerebral I/R. Targeting purinergic receptors, particularly those conserved and upregulated in response to stroke, may represent a promising therapeutic approach.

## 1. Introduction

Purinergic receptors, divided into the two main types P1 and P2, were first described by Burnstock in 1976; however, they were not widely recognized as legitimate receptors until the 1990s [1]. The purinergic P2 receptors are further classified into two subgroups: P2X and P2Y receptors. The seven P2X receptors (P2X1, P2X2, P2X3, P2X4, P2X5, P2X6, and P2X7) are ligand-gated ion channel receptors, while the eight P2Y receptors (P2Y1, P2Y2, P2Y4, P2Y6, P2Y11, P2Y12, P2Y13, and P2Y14) are G-protein-coupled receptors [1,2]. The gene sequences for all purinergic P2 receptors, except P2Y11, are available in rodents. Adenosine triphosphate (ATP) is a natural ligand for purinergic P2 receptors, and it also acts as a signaling molecule [2]. Purinergic signaling is implicated in a variety of physiological and pathophysiological processes, such as inflammation, pain, platelet aggregation, immune response, endothelial-mediated vasodilation, and cell death [1]. Because thrombosis, neuroinflammation, and cell death are central to ischemic stroke, purinergic signaling plays a critical role in its pathogenesis.

Ischemic stroke is a life-threatening emergency that occurs when blood flow to the brain is occluded by a blood clot, fatty plaque, or a dislodged plaque fragment within a cerebral artery. Ischemic stroke is the most common type that accounts for about 87% of all strokes [3]. Stroke is one of the leading causes of mortality and disability worldwide [4]. The only two FDA-approved recanalization treatments for ischemic stroke include thrombolysis with tissue-type plasminogen activator for clot dissolution and endovascular thrombectomy procedure for clot retrieval. Recanalization terminates ischemia and restores blood flow, which is known as reperfusion. Nonetheless, the brain damage inflicted during ischemia, along with the reperfusion injury that follows recanalization, contributes to the development of severe and long-lasting secondary effects, including additional brain injury and functional deficits. Despite years of intense research, treatments are not available to mitigate brain damage and enhance functional recovery following cerebral ischemia and reperfusion (I/R).

In response to an ischemic stroke, damaged or dying brain cells release excessive amounts of ATP, which overstimulates purinergic receptors on various cells within the ischemic brain, leading to further brain injury [5,6,7,8]. The primary cell types present in the ipsilateral brain following an ischemic stroke include neurons, astrocytes, microglia, oligodendrocytes, vascular endothelial cells, and infiltrating leukocytes. Nearly all these cells, along with platelets, express a wide variety of purinergic P2 receptors [9,10,11,12].

Preclinical studies have shown that increased purinergic receptor signaling in the brain following cerebral I/R can be both beneficial and detrimental, depending on experimental factors. These studies, conducted over the past several decades, have used both gene-deficient models and healthy rodents treated with agonists or antagonists of various purinergic P2 receptors. Consequently, targeting the appropriate receptor in a relevant preclinical model at the optimal time following cerebral I/R is essential for improving post-stroke outcomes.

Rats and mice are the two most commonly used species in preclinical stroke research. However, a critical gap remains in understanding species-specific differences in purinergic P2 receptor responses to ischemic stroke. A direct and systematic comparison of purinergic P2 receptor expression profiles over time across these species is lacking. This gap in our knowledge is significant because differences in receptor expression, temporal regulation, and signaling dynamics could influence how each species responds to ischemic injury, thereby impacting the reproducibility of biological responses and the translational relevance of preclinical findings.

To address this gap, the present investigation aimed to elucidate the temporal mRNA expression of all known purinergic P2 receptors in the ischemic brains of rats and mice. Understanding which receptors are upregulated or downregulated in each species following cerebral I/R and how these changes evolve over time is crucial for designing targeted studies and treatment strategies.

We hypothesize that purinergic P2 receptors would exhibit both conserved and species-specific expression patterns in the ischemic brain, reflecting underlying differences in post-stroke signaling between rats and mice. To test this hypothesis, we conducted a systematic analysis of purinergic P2 receptor mRNA expression in both species over a 7-day time course following cerebral I/R. This approach allowed us to identify receptors with consistent regulation across species, as well as those showing species-specific changes. Characterizing these expression profiles is essential for understanding species-dependent differences in purinergic signaling and for selecting receptor targets that are most likely to translate across preclinical models.

Our analysis was restricted to male animals to minimize biological variability and facilitate direct interspecies comparisons in the context of ischemic stroke. Sex-specific differences in purinergic P2 receptor expression have been previously reported. For example, Crain and colleagues found significant sex-related variations in basal purinergic P2 receptor expression in microglia from age-matched male and female mice across the lifespan [13]. Considering these established differences, we focused exclusively on species-specific differences between rats and mice. Additionally, the well-known neuroprotective effects of estrogen on ischemic stroke and the rapid hormonal fluctuations associated with the estrous cycle would introduce further complexity when comparing males and females, particularly in post-stroke time course studies [14,15,16,17,18]. To minimize these confounding factors, we limited the present study to male animals, enabling clearer interpretation of species-specific differences.

By clarifying both conserved and species-specific receptor responses, these insights will help researchers design future preclinical studies that better account for species differences, enhancing the relevance and translational value of these models for human stroke research. Ultimately, this research has the potential to advance therapeutic approaches for ischemic stroke, leading to improved patient outcomes.

## 2. Results

### 2.1. Baseline P2X and P2Y Receptor Expression: Variability and Correlation Between Species

**Figure 1:**
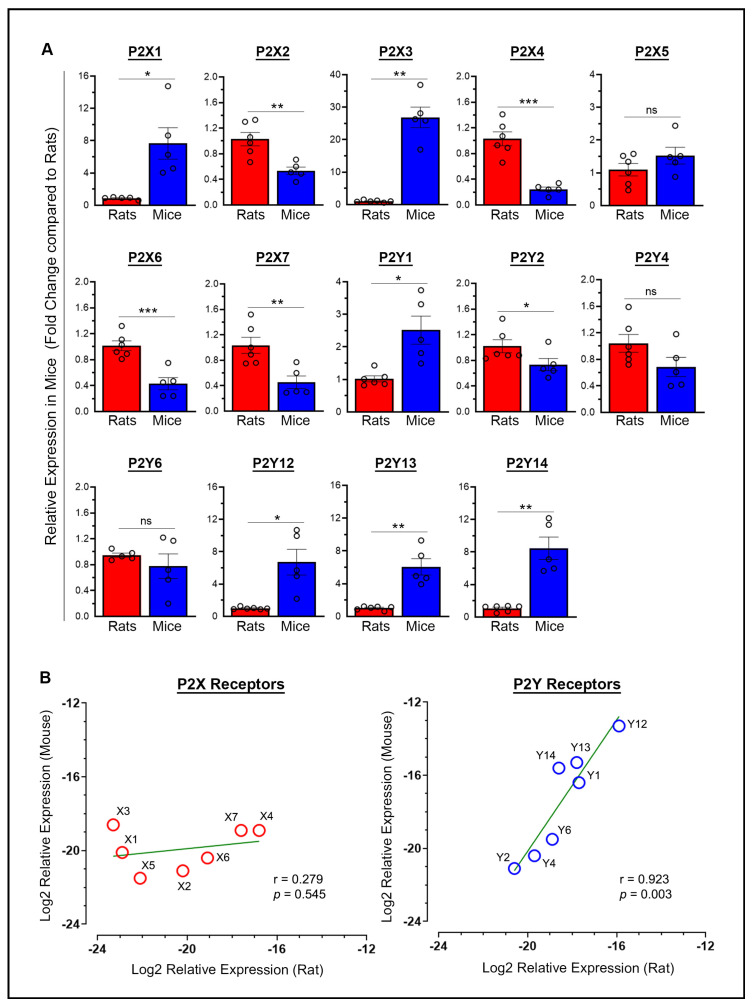
Relationship between the mRNA expression of purinergic P2X and P2Y receptors in the brains of rats and mice under baseline conditions. (**A**) The column scatterplots show the quantified baseline mRNA expression for various purinergic P2X receptors (P2X1 through P2X7) and P2Y receptors (P2Y1 through P2Y14) in mice expressed as fold change relative to their expression in rats. * *p* < 0.05, ** *p* < 0.01, *** *p* < 0.001. ns—not significant. (**B**) The scatterplots show the log2-transformed relative expression (group means; n = 5–6) for each of the studied P2X receptors (left panel) and P2Y receptors (right panel) in rats versus mice. In the plots, less negative log2 values (i.e., closer to −12) indicate higher expression, while more negative values indicate lower expression. The green line in each plot represents the line of best fit. Pearson’s correlation analysis of the log2-transformed relative expression values between rats and mice indicated no significant relationship for P2X receptors (r = 0.279, *p* = 0.545), but a strong positive linear relationship was observed for P2Y receptors (r = 0.923, *p* = 0.003). Baseline expression values in both species were obtained from sham animals euthanized on day 7 post-surgery.

The mean differences in baseline mRNA expression between rats and mice for each purinergic P2 receptor, with expression levels normalized to rats (taken as 1), were examined in the brains of sham rats and mice euthanized on day 7 post-surgery. Most of these receptors (9 out of 14, or 64%) showed approximately a 1-fold to 5-fold difference (P2X2, P2X4, P2X5, P2X6, P2X7, P2Y1, P2Y2, P2Y4, and P2Y6) (Figure 1A). Four additional receptors exhibited greater variability, with a 5-fold to 10-fold difference (P2X1, P2Y12, P2Y13, and P2Y14). In contrast, one receptor (P2X3) showed a variation exceeding 10-fold, with a 26.8-fold difference, which is considered well outside the typical range of biological variation. Statistically significant differences in the baseline mRNA expression of purinergic P2 receptors in mice versus rats were observed except for P2X5, P2Y4, and P2Y6 (Figure 1A). Significantly higher baseline mRNA expression was observed in mice compared to rats for P2X1 (*p* = 0.0253), P2X3 (*p* = 0.0012), P2Y1 (*p* = 0.0246), P2Y12 (*p* = 0.0233), P2Y13 (*p* = 0.007), and P2Y14 (*p* = 0.0043). In contrast, significantly lower expression was observed in mice compared to rats for P2X2 (*p* = 0.0038), P2X4 (*p* = 0.0004), P2X6 (*p* = 0.0008), P2X7 (*p* = 0.0066), and P2Y2 (*p* = 0.0455).

The range of ΔCt (target Ct minus Ct of the internal standard) values (group means) for the various P2X receptors, representing the highest and lowest expression levels, respectively, was 16.8 to 23.3 in rats, indicating a 90.5-fold difference, compared to 18.6 to 21.5 in mice, indicating a 7.5-fold difference. Similarly, the range of ΔCt values for the various P2Y receptors was 15.9 to 20.6 in rats, indicating a 26.0-fold difference, compared to 13.3 to 21.1 in mice, indicating a 222.9-fold difference. Thus, rats and mice showed variability in the expression levels of P2X and P2Y receptors, with rats showing greater variation in P2X receptors and mice in P2Y receptors. Furthermore, P2Y receptors were generally expressed at higher levels than P2X receptors in both rats and mice, with the P2Y12 receptor showing the highest expression among all P2 receptors in both species. Figure 1B shows scatterplots of log2-transformed ΔCt values, illustrating the relationship between the relative expression of P2X receptors (left panel) and P2Y receptors (right panel) in rats versus mice. Pearson’s correlation analysis of the log2-transformed relative expression values between rats and mice indicated no significant relationship for P2X receptors (r = 0.279, *p* = 0.545). However, a strong positive linear relationship was observed for P2Y receptors (r = 0.923, *p* = 0.003), indicating a similar expression pattern between rats and mice, despite variability among individual receptors. Overall, these results demonstrate that gene expression levels across purinergic P2Y receptors, but not P2X receptors, are closely related in both species, suggesting these receptors may play fundamental roles in shared physiological processes.

### 2.2. Similarities and Variations in Purinergic P2X Receptor Expression Following Stroke

**Figure 2:**
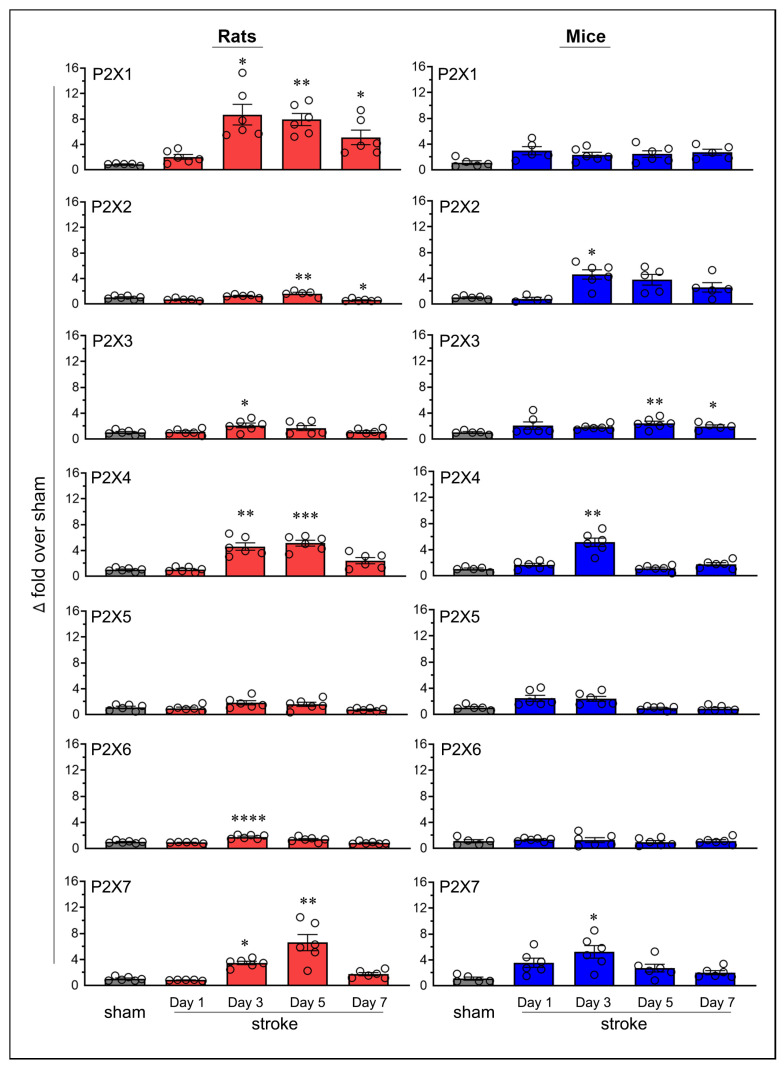
Temporal mRNA expression of purinergic P2X receptors in the ipsilateral brains of rats and mice following ischemic stroke. The column scatterplots show the quantified mRNA expression of purinergic P2X receptors (P2X1 through P2X7) as fold changes relative to day-7 sham in the ischemic brains of stroke-induced rats and mice. Animals were subjected to the right middle cerebral artery occlusion (2 h for rats and 1 h for mice) and euthanized at predetermined time points following stroke (days 1, 3, 5, and 7). * *p* < 0.05; ** *p* < 0.01; *** *p* < 0.001; **** *p* < 0.0001 vs. sham group.

Transient focal cerebral ischemia in rats and mice led to altered expression of purinergic P2X receptors in the ipsilateral brain (Figure 2). Sham-operated mice, euthanized on days 1, 3, 5, and 7, matching the time points used for stroke-induced animals, showed no statistically significant changes in P2X4 and P2X7 mRNA expression, indicating stable receptor expression over time (Figure S1). In contrast, stroke-induced animals exhibited robust and statistically significant increases in the expression of both receptors, as described below. Based on these findings, a single sham group (i.e., day-7 sham rats and mice) was used to calculate fold changes in mRNA expression for all purinergic P2X receptors in the ipsilateral brains of both stroke-induced rats and mice.

The Increased mRNA expression of the P2X1 receptor was statistically significant on post-ischemic days 3 (*p* = 0.016), 5 (*p* = 0.0025), and 7 (*p* = 0.0474) in rats; however, no statistically significant increases were observed in mice. The expression of P2X2 increased on day 5 (*p* = 0.0048) in rats and on day 3 in mice (*p* = 0.0153). P2X3 expression increased on day 3 (*p* = 0.0336) in rats and on days 5 (*p* = 0.0061) and 7 (*p* = 0.0447) in mice. The expression of P2X4 increased on days 3 (*p* = 0.0062) and 5 (*p* = 0.0004) in rats, and on day 3 (*p* = 0.0028) in mice. Neither rats nor mice showed statistically significant increases in P2X5 expression at any of the tested time points. P2X6 expression significantly increased on day 3 (*p* < 0.0001) in rats; however, no statistically significant increases were observed in mice. Finally, the expression of P2X7 increased on days 3 (*p* = 0.0204) and 5 (*p* = 0.0012) in rats, and on day 3 (*p* = 0.0217) in mice.

In addition to observing increases in mRNA expression, the P2X2 receptor showed a statistically significant decrease (*p* = 0.033, 42.9%) on day 7 in rats, with no similar decrease observed in mice. None of the other P2X receptors showed a decrease in expression at any time point in either species.

**Figure 3:**
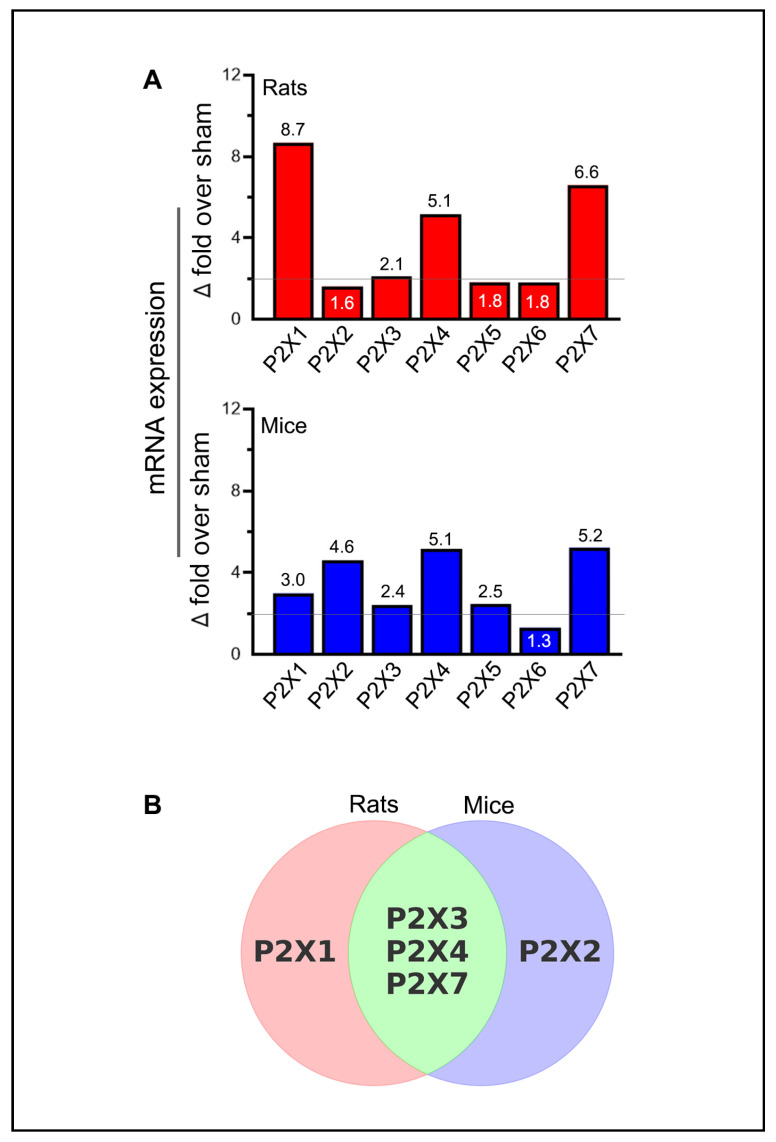
Summary of purinergic P2X receptor upregulation in rodents following ischemic stroke. (**A**) The bar graphs depict the highest observed mRNA expression levels (group means) for each purinergic P2X receptor at one of the tested time points in the ischemic brains of stroke-induced rats and mice. The horizontal lines in both bar graphs indicate the threshold above which increases in mRNA expression are considered biologically significant, provided they also reach statistical significance. (**B**) The Venn diagram illustrates the similarities and differences among the upregulated P2X receptors that demonstrate both biological and statistical significance at one or more of the tested time points (days 1, 3, 5, and 7 post-ischemia) in the ipsilateral brains of stroke-induced rats and mice.

Statistically significant increases in mRNA expression in stroke-induced animals are generally considered biologically significant if they exceed the sham group by at least two-fold. Thus, the increases in P2X2 and P2X6 in rats (1.6-fold and 1.8-fold above sham, respectively), while statistically significant, may not be biologically meaningful (Figure 3A). Notably, the increase in P2X1 expression (3-fold over sham) and P2X5 expression (2.5-fold over sham) in mice meets the threshold for biological significance but does not achieve statistical significance. The increased expression of P2X3, P2X4, and P2X7 receptors in both rats and mice, the P2X1 receptor in rats, and the P2X2 receptor in mice were both statistically and biologically significant (Figure 3B). Although not depicted, moderate decreases in mRNA expression, such as the approximately 50% reduction observed for the P2X2 receptor, may also hold biological significance.

### 2.3. Similarities and Variations in Purinergic P2Y Receptor Expression Following Stroke

**Figure 4:**
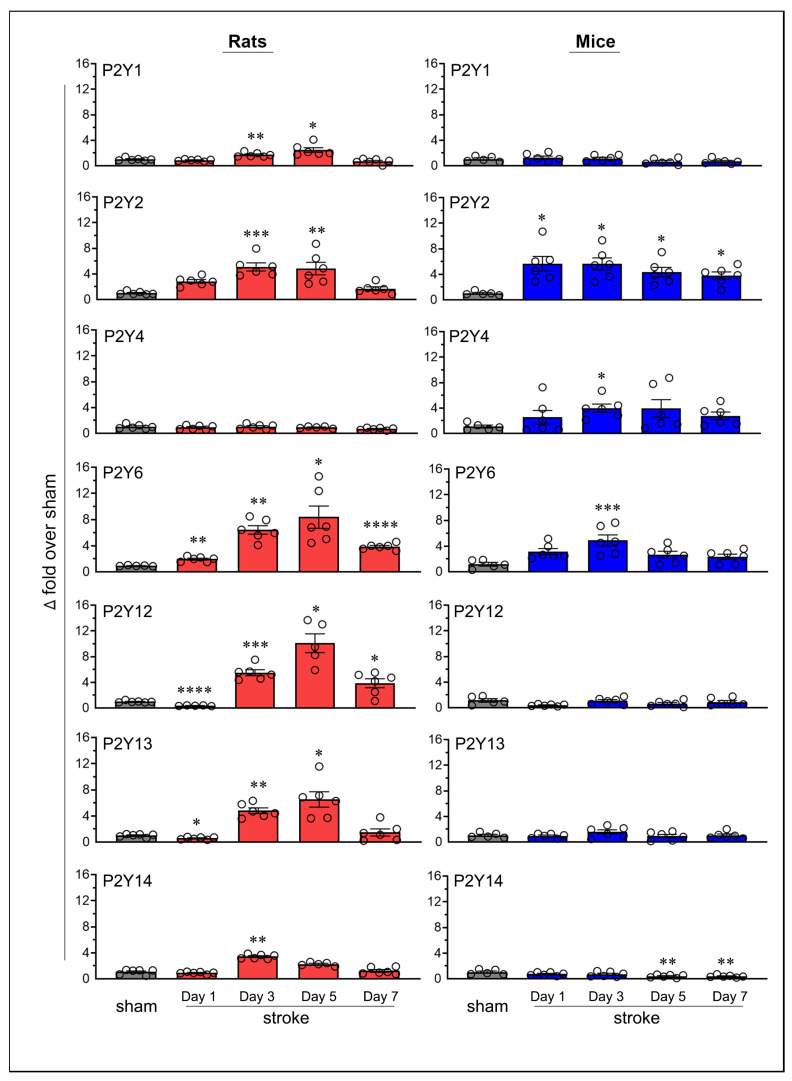
Temporal mRNA expression of purinergic P2Y receptors in the ipsilateral brains of rats and mice following ischemic stroke. The column scatterplots show the quantified mRNA expression of purinergic P2Y receptors (P2Y1 through P2Y14) as fold changes relative to day-7 sham in the ipsilateral brains of rats and mice. Animals were subjected to the right middle cerebral artery occlusion (2 h for rats and 1 h for mice) and euthanized at predetermined time points following stroke (days 1, 3, 5, and 7). * *p* < 0.05; ** *p* < 0.01; *** *p* < 0.001; **** *p* < 0.0001 vs. sham group.

Transient MCAO followed by reperfusion also altered the expression of P2Y receptors in the ischemic brains of both rats and mice (Figure 4). Since sham mice euthanized on post-surgery days 1, 3, 5, and 7 showed stable P2Y6 mRNA expression (similar to the pattern observed with P2X receptors), we used a single sham group (i.e., day-7 sham) in both species to compare changes in the mRNA expression of all P2Y receptors (Figure S1).

The mRNA expression of the P2Y1 receptor increased significantly in rats on post-ischemic days 3 (*p* = 0.0074) and 5 (*p* = 0.0297); however, no significant changes were observed in mice. P2Y2 receptor expression increased in rats on days 3 (*p* = 0.0002) and 5 (*p* = 0.002), and in mice on all tested days (*p* = 0.0344 on day 1, *p* = 0.0169 on day 3, *p* = 0.0239 on day 5, and *p* = 0.0109 on day 7). The P2Y4 receptor did not show statistically significant changes in rats; however, a significant increase was observed in mice on day 3 (*p* = 0.0193). P2Y6 expression increased significantly in rats on days 1 (*p* = 0.0041), 3 (*p* = 0.0014), 5 (*p* = 0.0238), and 7 (*p* < 0.0001) and in mice on day 3 (*p* = 0.0006).

In rats, P2Y12 expression increased on days 3 (*p* = 0.0007), 5 (*p* = 0.0109), and 7 (*p* = 0.032); P2Y13 expression increased on days 3 (*p* = 0.0012) and 5 (*p* = 0.0191); and P2Y14 expression increased on day 3 (*p* = 0.004). In contrast, no statistically significant increases were observed in the expression of P2Y12, P2Y13, or P2Y14 in mice.

In addition to increased receptor expression, several P2Y receptors also showed a decrease. Both P2Y12 and P2Y13 expressions decreased (*p* < 0.0001, 63.3% and *p* = 0.017, 42.3%, respectively) in rats on day 1 but remained unchanged in mice. For both receptors, this initial decrease was followed by a statistically significant increase on subsequent days, suggesting biphasic regulation in response to ischemic stroke. In mice, P2Y14 expression showed significant decreases on days 5 (*p* = 0.0055, 60.3%) and 7 (*p* = 0.0041, 62.3%), with no comparable decreases observed in rats. No other P2Y receptors exhibited a decrease in expression at any time point in either species.

**Figure 5:**
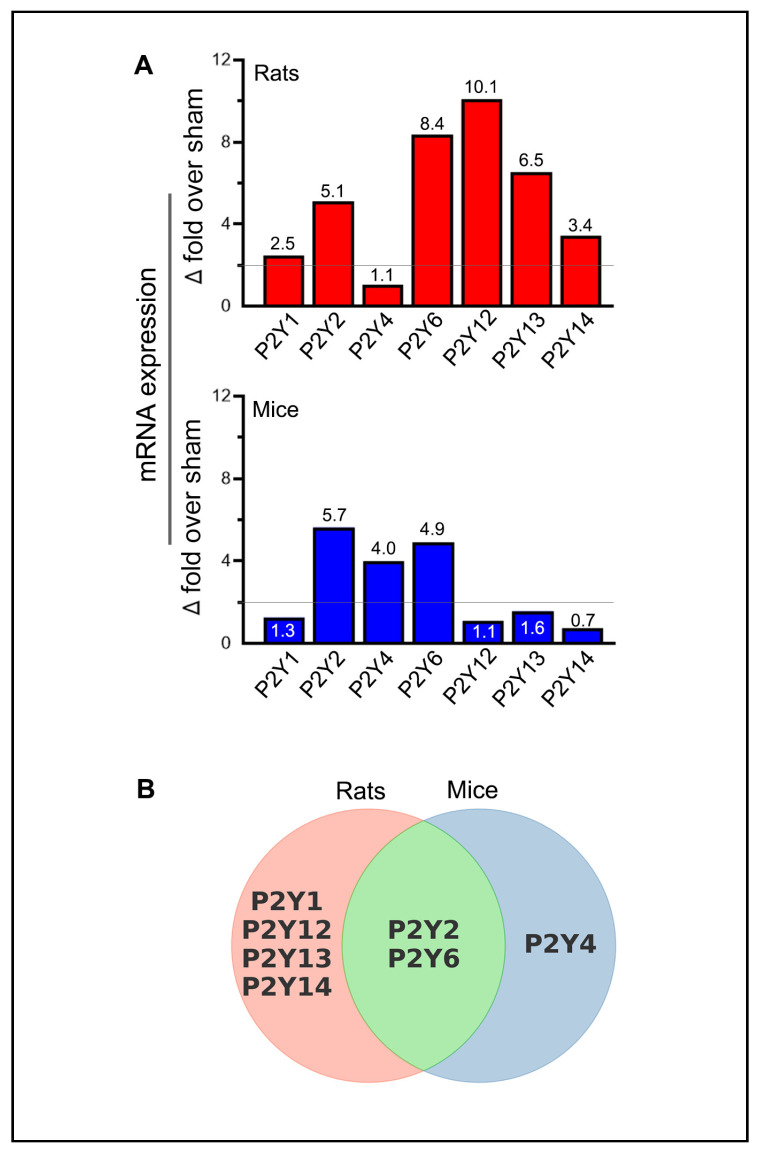
Summary of purinergic P2Y receptor upregulation in rodents following ischemic stroke. (**A**) The bar graphs depict the highest observed mRNA expression levels (group means) for each of the purinergic P2Y receptors at one of the tested time points in the ischemic brains of stroke-induced rats and mice. The horizontal lines in both bar graphs indicate the threshold above which increases in mRNA expression are considered biologically significant, provided they also reach statistical significance. (**B**) The Venn diagram illustrates the similarities and differences among the upregulated purinergic P2Y receptors that demonstrate both biological and statistical significance at one or more of the tested time points (days 1, 3, 5, and 7 post-ischemia) in the ipsilateral brains of stroke-induced rats and mice.

Unlike P2X receptors, all P2Y receptors that exhibited statistically significant increases in rats, mice, or both species also met the criteria for biological significance (Figure 5A). Additionally, several P2Y receptors exhibited decreases that were statistically significant and biologically relevant, particularly P2Y12 in rats and P2Y14 in mice. The increased expression of P2Y1, P2Y12, P2Y13, and P2Y14 receptors in rats, the P2Y4 receptor in mice, and the P2Y2 and P2Y6 receptors in both rats and mice were biologically and statistically significant (Figure 5B).

## 3. Discussion

Purinergic P2 receptors are of significant interest in ischemic stroke (as well as in traumatic brain injury and other neurodegenerative conditions) due to their roles in signaling and involvement in key cellular processes, including neuroinflammation, apoptosis, neurogenesis, and release of neurotrophic factors [19]. To determine which of these receptors may play a significant role in ischemic stroke, the present study explored the premise that purinergic P2 receptors would (1) exhibit upregulation or downregulation of mRNA expression following experimentally induced cerebral I/R, and (2) demonstrate similar changes in receptor expression in a second species, thereby validating the findings. In summary, this study examined the temporal mRNA expression of all purinergic P2 receptors with known gene sequences in the entire ipsilateral brains of rats and mice following transient focal cerebral I/R using the MCAO suture model. Specifically, the MCAO method and duration (2 h in young rats and 1 h in young mice) were standardized previously for each species to ensure appropriate, species-specific experimental conditions for obtaining moderate brain damage while minimizing mild or severe injury and mortality [20,21]. The overarching goal was to identify purinergic P2 receptors that could be targeted for the potential treatment of ischemic stroke, thereby supporting the development of future therapeutic strategies. Before examining these results, we first discuss our data on the relative expression levels of purinergic P2 receptors under baseline conditions to provide valuable context for understanding purinergic receptor mRNA responses following cerebral I/R. The baseline data were obtained from sham rats and mice that were euthanized on day 7 post-surgery to ensure a more complete recovery prior to analysis.

The relative expression of P2X and P2Y receptors in sham animals under baseline conditions varied considerably between species, with greater variation observed for P2X receptors in rats and for P2Y receptors in mice. Overall, P2Y receptors were expressed at higher relative levels than P2X receptors in both species. While no significant relationship was observed between rats and mice for P2X receptors, a strong positive linear relationship was found for P2Y receptors (Figure 1B), indicating that relative gene expression levels across P2Y receptors, but not P2X receptors, were closely linked in both species under baseline conditions. The majority of purinergic P2 receptors (64%) generally fell within the normal range of biological diversity (i.e., ≤5-fold). However, several receptors exhibited greater variability, with differences in the 5- to 10-fold range. Notably, the P2X3 receptor showed extremely high variation, reaching nearly 27-fold differences among rats, suggesting interspecies differences in receptor adaptation or functional roles. In our study, both rats and mice were healthy and of comparable age and weight within their respective species prior to surgery. Following ischemic stroke, body weight changes were observed in both species, but these changes were not drastically different between rats and mice. Therefore, we do not consider post-stroke weight loss to be a significant confounding factor in the animal weight or physiological characteristics to be significant confounding factors in the alteration of purinergic P2 receptor mRNA expression. Finally, in a separate experiment, the stability of mRNA expression was demonstrated in sham mice that were euthanized at the same timepoints as the stroke mice for several prominent purinergic P2 receptors (P2X4, P2X7, and P2Y6), reinforcing the reliability of using sham animals euthanized at any of the timepoints as controls for assessing changes in mRNA expression following cerebral I/R.

Following cerebral I/R, all purinergic P2 receptors examined exhibited significant upregulation of mRNA expression in at least one species (rats or mice), except for P2X5 and P2X6 receptors, which exhibited no upregulation in either species. This aligns with previous findings highlighting the distinct functional properties of these receptors. For example, P2X5 has been shown to exhibit lower responsiveness to ATP compared to all other P2X receptor subtypes, which may explain its unchanged expression following ischemic stroke [22]. Given its reduced responsiveness to ATP, it is likely that ATP release during the acute phase of ischemia has little to no effect on P2X5, allowing it to maintain baseline expression levels. Similarly, the unaltered expression of P2X6 after ischemic stroke is consistent with previous studies showing that oxygen and nutrient deprivation does not trigger activation or expression changes in the P2X6 receptor [23].

Five purinergic P2 receptors, P2X3, P2X4, P2X7, P2Y2, and P2Y6, were upregulated in both rats and mice following cerebral I/R. In contrast, five other receptors, P2X1, P2Y1, P2Y12, P2Y13, and P2Y14, were upregulated only in rats. Interestingly, 4 of the 5 receptors that were not upregulated in mice (P2X1, P2Y12, P2Y13, and P2Y14) showed relatively high variability in baseline receptor expression compared to rats, with differences exceeding 5-fold. A single receptor, P2Y4, was upregulated exclusively in mice following cerebral I/R. The magnitude of upregulation ranged from a minimum of 2.1-fold over sham for the P2X3 receptor in rats to a maximum of 10.1-fold over sham for the P2Y12 receptor in rats. Of note, the upregulation of the P2Y6 receptor in rats and the P2Y2 receptor in mice exhibited sustained upregulation throughout the study from post-stroke days 1 to 7, suggesting their significant involvement in the pathophysiological response to cerebral I/R. Previous research has associated the increased expression of P2Y6 with augmented vascular sensitivity to UDP-β-S after stroke [24]. Moreover, the sustained upregulation of the P2Y2 receptor observed in mice aligns with previous studies suggesting that IL-1β, oligomeric β-amyloid peptide, and other molecules can activate P2Y2 [25]. IL-1β, a pro-inflammatory cytokine, is often elevated in the acute phase following ischemic injury and plays a critical role in neuroinflammation [26]. The upregulation of P2Y2 receptors in response to IL-1β may enhance the cell’s ability to respond to extracellular nucleotides such as UTP, which can activate P2Y2 receptors and trigger downstream signaling pathways that promote cell survival [27]. This activation has been shown to have neuroprotective effects, such as reducing excitotoxicity and inflammation and improving mitochondrial function. The sustained activation of P2Y2 receptors by UTP during the post-stroke period likely contributes to neuroprotection by moderating inflammatory responses, stabilizing cellular homeostasis, and facilitating neuronal recovery [25]. Thus, the upregulation of P2Y2 receptors in the context of cerebral I/R may be a critical aspect of the neuroprotective mechanisms that help promote long-term neuronal survival.

Additionally, the upregulation of two receptors, P2Y12 and P2Y13, in rats was preceded by a significant decrease in mRNA expression, suggesting a biphasic regulatory mechanism. Compared to sham animals, the observed changes in purinergic P2 receptor expression in the ischemic brain following cerebral I/R could be largely attributed to neuroinflammation. This is because the primary sources of increased purinergic P2 receptor expression following cerebral I/R are activated glial cells, infiltrating monocytes, macrophages, and platelets that cross the disrupted blood-brain barrier into the ischemic brain [12,28]. To our knowledge, this is the first study to examine the gene responses of all purinergic P2 receptors in either rats or mice following cerebral I/R and represents the first comprehensive analysis of mRNA responses in both rats and mice using standardized ischemic stroke models tailored to each species.

The upregulation of purinergic P2 receptors represents a direct response of the brain to ischemia and reperfusion, which can either mitigate or exacerbate ischemic brain injury. These receptors may contribute to stroke pathology by triggering neuroinflammation and excitotoxicity or protect the brain by reducing cell death, enhancing tissue repair, and promoting recovery [29,30]. The conserved upregulation observed in this study for certain purinergic P2 receptors (P2X3, P2X4, P2X7, P2Y2, and P2Y6) across species suggests shared molecular pathways that are evolutionarily fundamental to the response to cerebral I/R. Specifically, the P2X4 receptor, which is abundantly expressed in vascular endothelial cells, has been implicated in neuroprotection through ischemic preconditioning, highlighting its critical role in modulating ischemic tolerance [31]. Similarly, P2Y2 has been shown to exert a neuroprotective role following nutrient and oxygen depletion by suppressing the YES-associated protein and subsequently hindering mitochondrial fission [32]. In contrast, the P2X7 and P2Y6 receptors have been linked to neuroinflammation after ischemic events, contributing to neuronal damage [32,33]. These findings underscore the potential of targeting purinergic receptors to mitigate the detrimental effects of cerebral I/R. Such conserved mechanisms may reflect critical processes that inform translational research and therapeutic strategies. In contrast, purinergic P2 receptors that did not show significant changes in mRNA levels in either rats or mice likely play a limited role in the overall response to cerebral I/R or may be regulated through alternative mechanisms.

Several purinergic P2 receptors have previously been identified as potential therapeutic targets for ischemic stroke, as demonstrated by improved stroke outcomes in diverse rodent stroke models following pharmacological inhibition or gene knockouts. These improvements include, but are not limited to, smaller infarct sizes, reduced blood-brain barrier disruption, decreased leukocyte infiltration, lower levels of pro-inflammatory mediators, and enhanced functional recovery. The identified receptors were P2X4 [8,31,34], P2X7 [35,36], P2Y1 [37,38], P2Y6 [39], and P2Y12 [40,41]. Notably, many of these studies (8 out of 10) were conducted in mice. Interestingly, in our study, only three of these receptors, P2X4, P2X7, and P2Y6, exhibited increased mRNA expression in mice. In contrast, all five of these receptors were upregulated in rats, with the P2Y1 and P2Y12 receptors being upregulated exclusively in this species. While these findings further support their potential roles in ischemic stroke mechanisms, they also highlight species-specific differences in their responses following cerebral I/R, which may hinder their translational potential from animal models to human therapies. This underscores the importance of identifying conserved responses across species to enhance the reliability of translational research.

The P2Y12 receptor is of particular interest in ischemic stroke pathology due to its dual role in microglial activation and platelet aggregation, and the clinical use of its antagonists for the prophylactic prevention of ischemic stroke. This receptor is primarily expressed on microglial cells in the brain and serves as a key target for therapeutic intervention [42]. Its activation leads to decreased cyclic AMP levels, which subsequently activate microglia, contributing to neuroinflammation and platelet aggregation during ischemic events [19]. P2Y12 receptor inhibitors, such as clopidogrel and ticagrelor, are clinically used for the secondary prevention of ischemic stroke due to their ability to reduce P2Y12-mediated platelet aggregation and provide neuroprotective effects [43,44]. In this study, the mRNA expression of the P2Y12 receptor significantly increased in rats from post-stroke days 3 to 7, following an initial decrease. The magnitude of this increase was the largest observed for any of the purinergic P2 receptors, making it even more surprising that P2Y12 expression remained unchanged in mice throughout the study following cerebral I/R, particularly given that both species exhibited their highest baseline expression for this receptor. The reasons behind this discrepancy, as well as other similar differences in the responses of some purinergic receptors between rats and mice, remain unclear. These discrepancies may involve changes in post-transcriptional or protein levels or reflect species-specific differences in gene regulation, inflammatory responses, or compensatory mechanisms.

This study has several limitations and caveats related to mRNA expression of purinergic P2 receptors and their biological relevance. First, changes in mRNA upregulation or downregulation do not always directly correspond to changes in receptor protein levels, as numerous factors can influence transcription and translation [45]. This point highlights the need for further investigation to validate these findings at the protein level and to assess receptor function in the context of cerebral I/R, providing a clearer understanding of their roles in stroke pathology and recovery. Additionally, the functional effects of changes in gene and protein expression levels may be offset or altered by various mechanisms, including ligand availability, receptor activation, receptor reserve, receptor internalization or trafficking, and compensatory actions by other receptors. Furthermore, the upregulation of purinergic P2 receptors following cerebral I/R, as often observed in this study, does not necessarily imply a deleterious role, as these receptors may mediate beneficial effects such as neuroprotective, anti-inflammatory, restorative, or pro-regenerative actions or even a combination of both detrimental and beneficial effects. Moreover, the lack of significant mRNA expression changes observed for some purinergic P2 receptors in the present study in both rats and mice does not necessarily rule out their involvement in ischemic stroke mechanisms. For instance, changes in protein expression, receptor activation, or downstream signaling pathways could still influence stroke pathology or recovery, suggesting that these receptors may still have therapeutic relevance. Finally, we were unable to study the P2Y11 receptor due to the absence of its gene sequence in rodents. This limitation is notable, as some evidence suggests that this receptor may play a role in ischemic stroke [19]. This receptor is expressed on human immune cells and is known to exert anti-inflammatory effects, which could influence stroke pathology [46].

In summary, while rats and mice share common purinergic P2 responses following ischemic stroke, they also exhibit species-specific differences that are critical for understanding how each organism adapts or responds to ischemic events at the molecular level. These differences include variability in baseline receptor expression and divergent changes following stroke, underscoring the importance of identifying purinergic P2 receptors with shared responses in both rats and mice when investigating their roles in ischemic stroke or other conditions. Receptors exhibiting shared responses across species are more likely to play critical roles in stroke mechanisms, as their conserved expression patterns suggest involvement in fundamental biological processes relevant across species and provide greater translational potential for therapeutic applications. In contrast, species-specific differences may provide insights into less conserved or adaptive pathways but are generally considered less indicative of a receptor’s central role in stroke pathology. The results of this study are extremely important and provide stroke researchers with essential guidance for selecting the appropriate species to test the efficacy of novel agents targeting purinergic P2 receptors, particularly those exclusively expressed in either rats or mice. Overall, our findings underscore the potential of certain purinergic P2 receptors as therapeutic targets for ischemic stroke.

## 4. Materials and Methods

### 4.1. Middle Cerebral Artery Occlusion (MCAO) and Reperfusion in Rats and Mice

**Figure 6:**
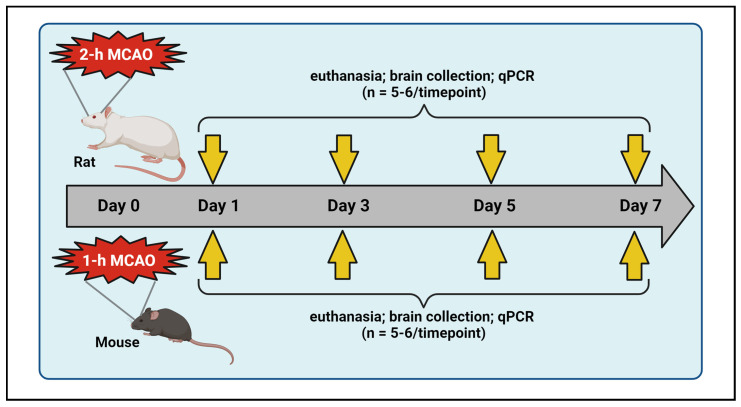
Schematic illustration of experimental design. Rats and mice were subjected to transient right middle cerebral artery occlusion (MCAO). Animals from designated experimental groups were euthanized at various time points (post-ischemic days 1, 3, 5, and 7), and their ipsilateral brains were collected and processed for real-time PCR analysis. This figure was created with BioRender.com (accessed on 29 September 2024) under a paid subscription.

A total of 40 healthy, young male Sprague-Dawley rats (2–3 months old) and 58 healthy, young male C57BL/6 mice (2–3 months old) were used in this study (Table 1). The rats and mice were obtained from Envigo Laboratories (Indianapolis, IN, USA) and the Jackson Laboratory (Bar Harbor, ME, USA), respectively. They were housed in dedicated animal rooms within the Laboratory Animal Care Facility at the University of Illinois College of Medicine Peoria (UICOMP) under controlled conditions, including a 12-h light/dark cycle, regulated temperature and humidity levels, and unlimited access to food and water. Both species were randomly assigned to either sham or stroke groups (Table 1). To induce transient focal cerebral ischemia followed by reperfusion, animals were subjected to an intraluminal suture MCAO, as previously described by our group [47]. Reperfusion was initiated by removing the suture after 2 h in rats and 1 h in mice. Post-surgical care was provided in accordance with the guidelines in “Ischemia Models: Procedural Refinements Of In Vivo Experiments” [48]. Animals were euthanized at various post-ischemic time points, and brain tissues were collected and stored at −70 °C until further analysis using quantitative real-time PCR (Figure 6). The study was conducted and reported in accordance with the guidelines stated in the “Animal Research: Reporting of In Vivo Experiments” [49].

### 4.2. Modified Neurological Severity Score (mNSS) Assessment in Rats

The mNSS is a composite of motor, sensory, reflex, and balance tests [50]. A score of 1–6 indicates mild injury, 7–12 indicates moderate injury, and 13–18 indicates severe injury. The mNSS assessment was conducted in rats 2–4 h post-MCAO and again on day 1 to determine the severity of stroke injury.

### 4.3. Neurological Deficit Score (NDS) Assessment in Mice

The NDS assessment (scoring: 0—no observable deficit; 1—forelimb flexion; 2—forelimb flexion and decreased resistance to lateral push; 3—forelimb flexion, decreased resistance to lateral push, and circling) was conducted in mice 2–4 h post-MCAO and again on day 1 to determine the severity of stroke injury.

### 4.4. Exclusion Criteria

Animals that either died or were euthanized during the study period, as well as those exhibiting postmortem bleeding in the region of the MCA, were excluded from the study. Additionally, rats with mNSS scores below 8 and mice with NDS scores below 2 were also excluded from the study. Finally, any data identified as outliers by the Grubbs’ test were excluded from the analysis.

### 4.5. RNA Isolation and cDNA Synthesis

Total RNA was extracted from the entire ipsilateral cerebral hemisphere, including the ischemic core, penumbra, and adjacent non-ischemic brain tissue, collected from the various experimental groups of rats and mice using TRIzol reagent (Invitrogen, Carlsbad, CA, USA). One microgram of total RNA from each sample was reverse transcribed to cDNA using the iScript cDNA Synthesis Kit (Bio-Rad Laboratories, Hercules, CA, USA), and the resulting cDNA was stored at −20 °C for subsequent analysis.

### 4.6. Quantitative Real-Time PCR Analysis

Real-time PCR analysis was conducted using the SYBR Green method. For each diluted (1:10) cDNA sample, the reaction mixture was prepared using iTaq Universal SYBR Green Supermix (Bio-Rad Laboratories, Hercules, CA, USA) according to the manufacturer’s instructions. Forward and reverse primers for the target genes, sourced from Integrated DNA Technologies (Coralville, IA, USA), were diluted 1:10 in nuclease-free water, with primer sequences listed in Table 2. The PCR cycle conditions were as follows: an initial denaturation at 95 °C for 5 min, followed by 40 cycles of 95 °C for 30 s, 58–62 °C for 30 s, and 72 °C for 30 s, and a final extension at 72 °C for 5 min. The reactions were conducted on an iCycler IQ (Multi-Color Real-Time PCR Detection System; Bio-Rad Laboratories, Hercules, CA, USA).

Data was collected using CFX Manager software Version 3.1 (Bio-Rad Laboratories, Hercules, CA, USA) and expressed as threshold cycle (Ct) values, representing the number of cycles required for the fluorescent intensity of SYBR Green dye to exceed the background fluorescence, which is inversely related to the quantity of the target nucleic acid in the sample. Specific amplification of the target gene was confirmed by a single, uniform melt curve peak at the expected melting temperature for each target gene. *18S* rRNA served as internal control, and relative quantification of target gene expression was normalized to *18S* rRNA. The fold change in target gene expression in the test sample relative to the control sample was calculated using the formula 2^−ΔΔCt, where ΔΔCt = ΔCt of test − ΔCt of control.

### 4.7. Statistical Analysis

Statistical analyses were conducted using GraphPad Prism 8.4.3 for Windows. Quantitative data was screened for outliers using the Grubbs’ test followed by tests for normality and equality of variances. Based on the results of these tests and the number of groups present, the appropriate statistical tests were applied. For comparisons involving only two groups, a two-tailed, unpaired Student’s *t*-test (parametric) or Mann-Whitney U-test (non-parametric) was used. For multiple-group comparisons, parametric analyses included one-way ANOVA followed by Dunnett’s or Tukey’s multiple comparison tests. When assumptions of homogeneity of variances were violated, the Brown-Forsythe or Welch ANOVA was used, followed by Dunnett’s T3 multiple comparison test. Non-parametric analyses included the Kruskal-Wallis test followed by Dunn’s multiple comparison test. In some instances, Pearson’s correlation coefficient was used to assess the relationship between rats and mice in the baseline expression of P2X and P2Y receptors. Differences between groups were considered significant at *p* < 0.05. All data are reported as mean ± SEM.

## Figures and Tables

**Table 1:** Experimental groups and animal usage.

Group Name	Group Description	Number of Animals		
		For Real Time PCR	Excluded	Total
Mice				
Sham: Mice subjected to surgery without intraluminal monofilament suture insertion to simulate stroke conditions				
	- Sham mice euthanized on day 1 post-surgery	6♂	0	6♂
	- Sham mice euthanized on day 3 post-surgery	6♂	0	6♂
	- Sham mice euthanized on day 5 post-surgery	5♂	0	5♂
	- Sham mice euthanized on day 7 post-surgery	5♂	0	5♂
Stroke: Mice subjected to 1-h MCAO				
	- Stroke mice euthanized on day 1 post-MCAO	6♂	2♂	8♂
	- Stroke mice euthanized on day 3 post-MCAO	6♂	3♂	9♂
	- Stroke mice euthanized on day 5 post-MCAO	6♂	4♂	10♂
	- Stroke mice euthanized on day 7 post-MCAO	6♂	3♂	9♂
Rats				
Sham: Rats subjected to surgery without intraluminal monofilament suture insertion to simulate stroke conditions				
	- Sham rats euthanized on day 7 post-surgery	6♂	0	6♂
Stroke: Rats subjected to 2-h MCAO				
	- Stroke rats euthanized on day 1 post-MCAO	6♂	2♂	8♂
	- Stroke rats euthanized on day 3 post-MCAO	6♂	2♂	8♂
	- Stroke rats euthanized on day 5 post-MCAO	6♂	3♂	9♂
	- Stroke rats euthanized on day 7 post-MCAO	6♂	3♂	9♂

MCAO, middle cerebral artery occlusion; PCR, polymerase chain reaction.

**Table 2:** Primers used for real time PCR analysis.

Gene	NCBI Reference Sequence	Primer Sequence		Amplicon Length (bp)
		Forward (5′-3′)	Reverse (5′-3′)	
Rat				
P2X1	NM_012997	ttgtgcagagaacccagaag	acagttgcctgtgcgaata	100
P2X2	NM_053656	gtgacctggacttgtctgaat	tggtggtagtgccgtttatc	135
P2X3	NM_031075	gtcatggacgtgtcggattat	cagcggtacttctcttcattct	128
P2X4	NM_031594	caatgtgtctcctggctacaa	ttcccagcctttccaaaca	129
P2X5	NM_080780	gaatgggactgtgaccttgat	gtaatacctggcgaacctgaa	126
P2X6	NM_012721	acctgggacaacacctatttc	agcaatgcaaggtcctcaa	125
P2X7	NM_019256	tttggccaccgtgtgtatt	gatgggctcacacttctttct	139
P2Y1	NM_012800	tttgtatgtgctcaccctacc	agatgctgccatagaggtttac	125
P2Y2	NM_017255	tgccgctgctggtttatta	gatgctgcagtagaggttagtg	107
P2Y4	NM_031680	tcgatttgcaagccttctct	catggcacaggatggtagtt	113
P2Y6	NM_057124	cctcttctatgccaacctacac	agccaaacgactccacatac	147
P2Y12	NM_022800	ctgaagaccaccagaccattta	cctcctgttggtgagaatcat	129
P2Y13	NM_001002853	ggcatcaaccgtgaagaaatg	tcttggcaatcaccgtgtaa	140
P2Y14	NM_133577	catggccataacgaggaagat	gcacgaaacagacgacaaatac	125
*18S* rRNA	NR_046237	acgtctgccctatcaactttc	ttggatgtggtagccgtttc	117
Mouse				
P2X1	NM_008771	ttgtgcagagaacccagaag	acagttgcctgtgcgaata	100
P2X2	NM_001164834	gggtcatcatcaactggaac	aagaggcagggtcatactt	102
P2X3	NM_145526	gaagggaaacctccttcctaac	aatcctgcccagcaaactta	125
P2X4	NM_001310720	ctcatcctggcttacgtcatt	agctgagaagtgttggtcac	125
P2X5	NM_033321	cacttcagctccaccaatct	atcaaggtcacagtcccattc	138
P2X6	NM_001159561	tgtcatcaccaaactcaaggg	ggaagttggttaccaggaagaa	128
P2X7	NM_001038887	aagtgggtcttgcacatgat	cacctctgctatgcctttga	126
P2Y1	NM_001282016	ggcaggctcaagaagaagaa	tcattggacgtggtgtcatag	146
P2Y2	NM_001302347	ccgagagctctttagccattt	ggccataagcacgtaacaga	103
P2Y4	NM_020621	tctgcctgaggagtttga	atgagtccatagcagacca	102
P2Y6	NM_183168	ggcttgttattgtcgcatgg	aggaagctgatggcaaaga	132
P2Y12	AJ312130	gaagaccaccaggccattta	cctcctgttggtgagaatcat	127
P2Y13	NM_028808	tcacacctgccagttcattt	cctgctgtccttactcctaaac	115
P2Y14	NM_133200	cgtgttgtacggtatggtctt	gtcagccaccactatgttctt	124
*18S* rRNA	NR_003278	tgagaaacggctaccacatc	gcctcgaaagagtcctgtatt	106

## Data Availability

All the data for this study will be made available at reasonable request to the corresponding author.

## Supplementary Materials

The supporting information can be downloaded at: https://www.mdpi.com/article/10.3390/ijms26062379/s1.
