# Supplementary material for: Temporal mRNA Expression of Purinergic P2 Receptors in the Brain Following Cerebral Ischemia and Reperfusion: Similarities and Distinct Variations Between Rats and Mice
Source: Int J Mol Sci. 2025 Mar 7;26(6):2379. doi: 10.3390/ijms26062379 (PMC11941906; doi:10.3390/ijms26062379)
Supplement: Supplementary file 1 [file ijms-26-02379-s001.zip › ijms-3469378-supplementary.pdf]

Supplementary Material

Temporal mRNA Expression of Purinergic P2 Receptors in the Brain Following Cerebral Ischemia and Reperfusion: Similarities and Distinct Variations Between Rats and Mice

Siva Reddy Challa [1,†], Hunter Levingston [1,†], Casimir A. Fornal [1], Isidra M. Baker [1], Joseph Boston [1], Nidhi Shanthappa [1], Pavani Unnam [1], Jeffrey D. Klopfenstein [1,2,3], Krishna Kumar Veeravalli [1,2,4,5,*]

[1] Department of Cancer Biology and Pharmacology, University of Illinois College of Medicine Peoria, Peoria, IL 61605, USA

[2] Department of Neurosurgery, University of Illinois College of Medicine Peoria, Peoria, IL 61605, USA

[3] Illinois Neurological Institute, OSF HealthCare, Peoria, IL 61603, USA

[4] Department of Pediatrics, University of Illinois College of Medicine Peoria, Peoria, IL 61605, USA

[5] Department of Neurology, University of Illinois College of Medicine Peoria, Peoria, IL 61605, USA

* Correspondence: krishnav@uic.edu

† These authors have contributed equally to this work.

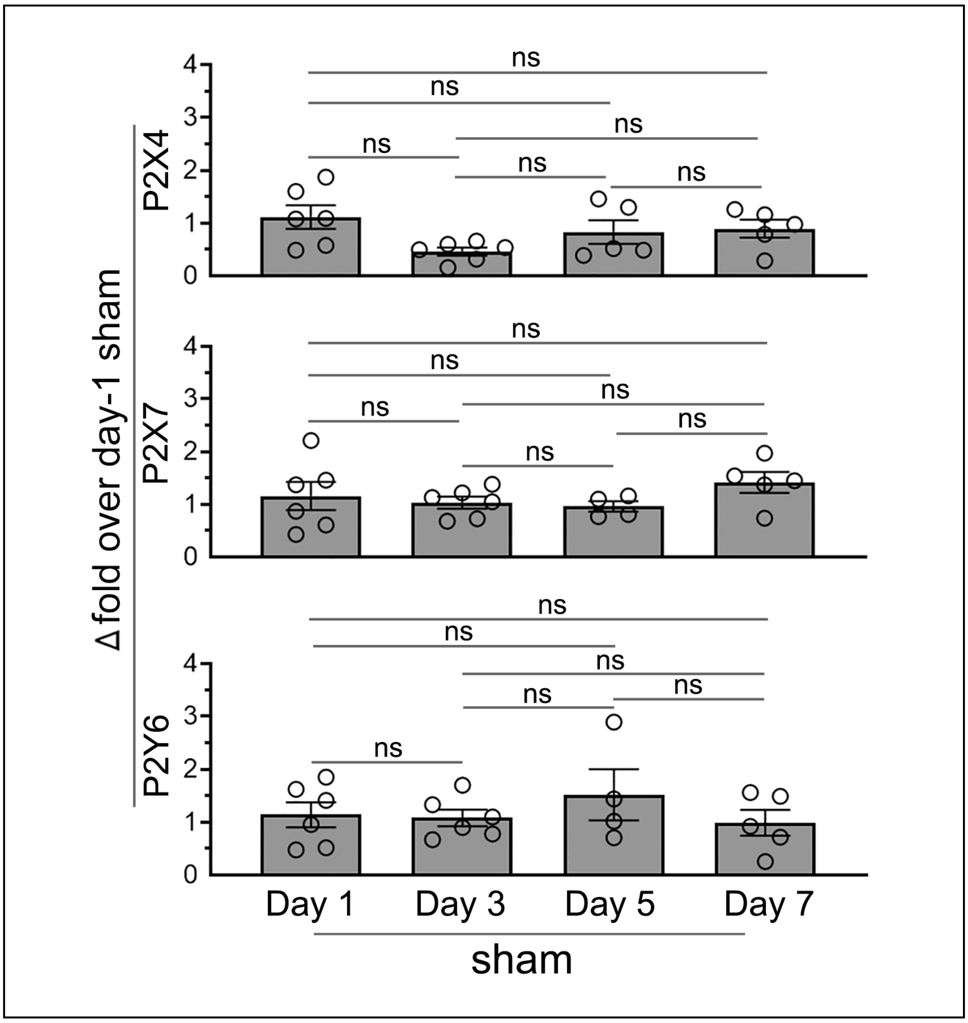

Figure S1. The mRNA expression of purinergic P2 receptors in sham animals euthanized at various time points after surgery showed no significant changes, indicating stability of receptor expression. The column scatterplots depict the quantified mRNA expression of selected purinergic P2 receptors (P2X4, P2X7, and P2Y6) as fold changes relative to day-1 sham. Samples were obtained from the ipsilateral brains of sham-operated mice euthanized on days 1, 3, 5, and 7 after surgery. In contrast to sham animals, these receptors demonstrated significant upregulation in stroke-induced animals, highlighting their potential involvement in ischemic responses. ns, not significant.
